# Vegan food geographies and the rise of Big Veganism

**DOI:** 10.1177/03091325211051021

**Published:** 2022-01-29

**Authors:** Alexandra E Sexton, Tara Garnett, Jamie Lorimer

**Affiliations:** Department of Geography, 7315 University of Sheffield, Sheffield, UK; TABLE, School of Geography and the Environment, 6396 University of Oxford, Oxford, UK; School of Geography and Environment, 6396 University of Oxford, Oxford, UK

**Keywords:** Vegan, food geography, alternative food networks, plant-based, mainstream

## Abstract

Veganism is the subject of an increasingly diverse body of social scientific research, yet it remains relatively understudied in geography. Meanwhile, contemporary cultural commentaries note how veganism has gone mainstream, with critics warning of veganism’s corporate nature – expressed in the rise of what we term ‘Big Veganism’. We argue that food geographers are well placed to examine these trends. We first review vegan studies work beyond geography that examines and critiques the mainstreaming of veganism. We focus on literature that explores *multiple contested modes* of veganism, veganism as *praxis in place* and the rise of *corporate veganism* as useful foundations for geographers to build on, particularly in light of currently unfolding developments in vegan cultures and practice. Taking this work forward, we identify four conceptual traditions from research in food geographies – *following foodways*, *alternative food networks* and the *cultural* and *material politics* of eating – to develop a ‘vegan food geographies’ programme that aims to advance critical geographic work on veganism and the emerging implications of its contemporary mainstreaming.

## I The rise of Big Veganism

In a preview of global market trends, *The Economist* declared that ‘2019 will be the year veganism goes mainstream’ (Parker, 2018). This prophesy of the ‘year of the vegan’ was echoed across international business and cultural commentary, with articles offering tips on how to launch vegan start-ups and how to ‘veganise’ existing companies (Fox, 2017; Cook, 2019). The previous 12 months had seen unprecedented growth in sales of vegan products in North America and Europe. In 2018, the US retail market for plant-based foods grew by 20% to total $3.3 billion in sales (PBFA, 2018). In the UK, approximately 600,000 people identified as ‘vegan’ and a record number of people (250,000) reportedly took the Veganuary pledge to go vegan throughout January in 2019 (Smithers, 2019). UK high street retailers from Waitrose to Greggs launched their own vegan product ranges (Calnan, 2019; Monaghan, 2019) and vegan brands were some of the biggest acquisitions by global livestock product companies (e.g. The Vegetarian Butcher by Unilever (Michail, 2018; see also Good Food Institute, 2019). Online, #vegan and #plantbased have become leading food hashtags on Instagram (used in 115 million and 36.3 million posts, respectively, at time of writing). Vegan celebrities (Lewis Hamilton, Arianna Grande) and vegan influencers (Deliciously Ella, BOSH!) are becoming a lucrative element of the cultural economy of food (Goodman and Jaworska, 2020).

While it is important to keep these economic trends in perspective with the continued dominance of global animal food systems, they reflect a discernible shift in the historical position of veganism at the margins of Western culture. Veganism and vegetarianism are some of the oldest ‘alternative’ practices in meat-centred or ‘carnist’ (Joy, 2011) food cultures (Spencer, 1996; Iaccobo and Iaccobo, 2004), with well-documented connections to Victorian programmes of religious reform and women’s suffrage (Leneman, 1999; Maurer, 2002), and more recent countercultural, anarchist, Rastafarian and alt-right movements (Forchtner and Tominc, 2017; Waldstein, 2016; Werkheiser, 2013; White, 2017). While vegans have often faced ridicule and exclusion (Cole and Morgan, 2011; Doyle, 2016), recent stories portray veganism as exciting and hopeful (Pendergrast, 2016). Veganism has become cool: an aspirational lifestyle for younger, urban and wealthier demographics (Nguyen, 2017). Veganism is also increasingly presented by scientists and in mainstream media as the sensible antidote to the health impacts of meat consumption, and to the ecological impacts of conventional livestock production (Godfray et al., 2018; Poore and Nemecek, 2018).

Enthusiasts suggest that this mainstreaming of veganism has the potential to enact significant ethical, ecological and health changes to an agri-food system increasingly understood as broken (Friedrich, 2020; Dutkiewicz and Dickstein, 2021). Meanwhile critics caution that this mainstreaming risks diluting the radical ethics of veganism, and argue that it is characterised by notable continuities and consolidations of who is developing, producing and profiting from new vegan products (White, 2018; Clay et al., 2020). These debates centre on the emergence of what we term ‘Big Veganism’: the recent turn by ‘Big Food’ and ‘Big Agriculture’ (Stuckler and Nestle, 2012) to veganise their offerings and bring vegan products into mainstream spaces of food production and consumption. This model of veganism’s mainstreaming is evidenced by the kinds of developments described at the opening of this paper that have seen the biggest names in food and agriculture over recent years increasingly incorporate vegan options into their operations through direct investment, acquisitions and even corporate rebranding.^1^ Regular headlines attest to global networks of commodity plant-based (e.g. soya, wheat, pea) and biotechnological ingredients (Stephens et al., 2018) being mobilised by agri-businesses to meet the growing demand for high-tech, ersatz, ‘ultra-processed’ foods, like the flagship Impossible Burger and plant-based milks (Mylan et al., 2019; Sexton, 2018; Clay et al., 2020; Tziva et al., 2020).

This model is grounded in the prevalent neoliberal politics of individual food choice (White, 2018) and carnist food cultures (Joy, 2011), in which fetishized, often expensive products are marketed primarily to privileged audiences that celebrate white ‘shredded’ bodies and the welfare of charismatic animals (Harper, 2012; Wright, 2015; Doyle, 2016). Alternative versions of lower-tech, minimally-processed and socially embedded vegan foodways are noticeably absent from the Big Vegan model. While examples of these alternative vegan offerings can be found in more niche food and health retailers (e.g. Hodmedod’s Pulses and Grains, and Riverford Organic in the UK), Big Veganism has arguably emerged as the significant driving force of the current mainstreaming of vegan identity, practice and products in Europe and North America. The considerable cultural and financial power it continues to amass at pace is defining the politics of possibility (Guthman, 2008) of what contemporary veganism is, who it is for, the geographies and scales it encompasses, and what kinds of alterity to conventional food systems it can deliver (White, 2018).

These trends in vegan mainstreaming and its possible futures raise important questions for food geographers. Geography ought to be well placed to explore such developments given the longstanding history of work on alternative food networks and their mainstreaming (Guthman, 2003; Watts et al., 2005; Harris, 2009; Goodman et al., 2012; Slocum and Cadieux, 2015). But veganism is largely absent from geographic enquiry, with a few notable exceptions in work on animal geographies (Twine, 2014a), radical geographies (Véron, 2016; White, 2017) and alternative food economies (Hahn and Bruner, 2012), including a nascent programme for a new field of ‘vegan geographies’ (Hodge et al., forthcoming). A recent collaborative paper by Morris et al. (2021) outlines a timely and comprehensive social science and humanities research agenda for studying the ‘challenges of moving beyond animal-based food systems’. The authors note the disparate nature of existing research on the cultures, practices, politics and ethics of eating animals, and within this corpus highlight that work on vegetarianism and veganism has been largely consumption-oriented (ibid, 2). We observed a similar orientation in our search of literatures on vegan mainstreaming. We found most of this work largely concentrated in the interdisciplinary field of vegan studies (Wright 2015), which has remained mostly separate from core food geography debates. In this article, we identify avenues for how geography can both engage with and extend this existing work – particularly by drawing on conceptual traditions in food geography that view production and consumption as mutually co-constitutive (Lockie and Kitto, 2000; Goodman D., 2004; Kneafsey et al., 2021). We build directly on Morris et al. (2021) and Hodge et al.’s (forthcoming) agenda-setting work by exploring what geography, and *food* geography more specifically, can offer research on how transitions away from animal-based food systems are currently being framed and materialised. We refer to this complementary research programme as *vegan food geographies*.

We begin by reviewing the extensive scholarship on veganism beyond the admittedly porous disciplinary boundaries of geography, focussing on work that explores and critiques the mainstreaming of veganism in Western Europe and North America. This literature identifies multiple contested modes of veganism, explores veganism as praxis in place and cautions against the rise of corporate veganism. This work provides useful foundations for geographers to build on, particularly in light of currently unfolding developments in vegan cultures and practice. The unprecedented speed and scale of vegan mainstreaming in recent years calls for more critical engagement with the current shifts in political economic, material, cultural and moral structures of vegan foodways. As we and others identify (e.g. Morris et al., 2021), a key component of this requires a broadening of the consumption-oriented nature of much previous vegan studies work. The following sections explore how vegan scholarship might be extended in these ways through engagement with the conceptual and empirical concerns of food geographies, focussing on work that: follows and places foods; critically examines the alterity of food networks, and explores the cultural and material politics of eating. We draw these literatures together in conclusion to outline future pathways of vegan food geographies research that further engages geographic work on food with vegan scholarship and practice. Within this broader agenda, we highlight the emergence of Big Veganism as a particularly timely subject for geographers to critically engage with given its growing prominence in contemporary foodways, and its implications for the spatial, political economic and cultural (re)ordering of global food networks.

## II Vegan studies and the politics of mainstreaming

Veganism as ethics and social practice has been the subject of an increasingly formalised and diverse body of social science research. History (Spencer, 1996; Leneman, 1999; Iacobbo and Iacobbo, 2004), critical race and gender studies (Harper, 2010; Polish, 2016; Brueck and McNeil, 2020), cultural theory (Adams, 1990; Giraud, 2021; Wright, 2015), sociology (Cherry, 2006; Greenebaum, 2012; Wrenn, 2017), media and communication studies (Cole and Morgan, 2011; Doyle, 2016), philosophy (Francione, 2012), market studies (Fuentes and Fuentes, 2017) and psychology (Sneider and Te Molder, 2004) are just some of the multi-disciplinary strands that make up an emerging field of ‘vegan studies’ (Wright, 2015). While a more exhaustive review is beyond the scope of this paper (see Giraud, 2021 for an in-depth history of vegan academic-activist traditions), we focus here on three key strands of this work as a useful starting point for understanding the contemporary vegan moment.

### 1 Multiple veganisms

Recent work in vegan studies has sought to map and critique the diversity of claims and practices that get subsumed by the label veganism. The UK Vegan Society defines veganism as:

A philosophy and way of living which seeks to exclude – as far as is possible and practicable – all forms of exploitation of, and cruelty to, animals for food, clothing or any other purpose; and by extension, promotes the development and use of animal-free alternatives for the benefit of animals, humans and the environment. In dietary terms it denotes the practice of dispensing with all products derived wholly or partly from animals.

While this definition may seem familiar and uncontroversial to readers of this journal, it emerges from, and seeks to bridge, longstanding philosophical and political deliberations within vegan scholarship and activism over what constitutes authentic vegan praxis (Greenebaum, 2012). There are a broad range of definitions, motivations and everyday practices amongst vegan communities in the UK and US (Cherry, 2006) and this diversity leads to debate. For example, as Greenebaum notes, the identity markers of ‘health’, ‘environmental’ and ‘ethical’ vegan – and more recent labels like ‘vegan curious’, ‘flexitarian’ and ‘plant-based’ – are ‘not merely descriptive differences; they are value-laden within the vegan community’ (2012, 130).

Health veganism has historically proven more effective at achieving widespread public acceptance in the US (Maurer, 2002). Moreover, recent market data shows that concerns for personal health and the environment rather than animal welfare are driving recent vegan adoption in the UK and US (Shoup, 2019; Pendergrast, 2016). While some scholars and activists welcome any reductions in animal-based consumption regardless of motivation, finding hope in the prospect of more quickly reducing the systemic harms of factory farming through veganism’s partnerships with agri-food corporates and high-tech start-ups (Friedrich, 2020; Dutkiewicz and Dickstein, 2021), others argue that veganism should not be divorced from animal rights. They strongly advocate for a universal vegan ethic of total animal liberation (Regan, 1983; Francione, 2012). For vegan writer Gary Francione (2016, np), veganism is ‘first and foremost about nonviolence to other sentient beings’. As an advocate for a universal abolitionist approach, he sees veganism as a choice that all persons ‘can make today – right now – if we believe that animals matter morally’ (ibid). He expresses the shared concern of vegan abolitionists that the mainstreaming of veganism is leading to the dilution and splintering of authentic vegan praxis.

Yet the quest for an authentic vegan ethics based on the single issue of animal rights or animal welfare has been questioned in recent work in vegan studies. Drawing on ecofeminist and anarchist theories, critical vegan scholars have challenged the privilege bound up in universal abolitionist perspectives. For example, Warkentin (2012, 500) denounces the universal prescription of a ‘pure’ vegan lifestyle, arguing that a democratic vegan ideology and scholarship should allow ‘for rigorously considered grays rather than demanding all-encompassing black or white positions’. Similarly, Twine notes that ‘there is much diversity within veganism’ in terms of motivations and practice, and suggests that ‘it should not be assumed simply that [veganism] is always a choice for non-violence’ (Twine, 2014b, 192). His argument builds directly on the influential work of Adams (1990); Harper (2010, 2011, 2012) and others who spotlight the power relations within mainstreamed vegan praxis that intersect class, race and gender, with concerns for ecologies and different species (Hamilton, 2016; Polish, 2016; Gaard, 2017).

The intellectual activism in this work has been instrumental in pushing the storying of veganism backwards in time to challenge the primacy often afforded to the founding of the UK Vegan Society in 1944 (Brueck and McNeil, 2020). It also aims to look outwards to encompass a broader diversity of cultural, socioeconomic and gendered practices of veganism. For example, critics of the white aesthetics and politics of mainstream veganism advocate for ‘race consciousness’ (Harper, 2012) and more intersectional approaches to vegan scholarship to redress the erasure of underrepresented voices – particularly people of colour – from the intellectual traditions of veganism (Harper 2010). They aim to push at the boundaries of what authentic vegan practice looks like in order to legitimise more pluralistic models of vegan eating.

### 2 Veganisms as praxis in place

Vegan activists and vegan studies scholars have long explored veganisms as praxis in place (White and Cudworth, 2014; Doyle, 2016; Véron, 2016; Martin, 2019). A recurring methodological approach is to offer first-hand researcher testimonies of the affective, physical places of farm animal life, including livestock auction rooms (Gillespie, 2018), slaughterhouses (Lockwood, 2018) and farm animal sanctuaries (Tulloch, 2018). These accounts intentionally pay attention to the emotional responses of the witness (the researcher) and the witnessed (the animals), with particular emphasis on grief, fear and anger (Oliver 2020). Gillespie (2018) notes that witnessing has been a longstanding format of animal activism – for example, through vigils and undercover videos. As an academic method, witnessing is similarly political in making visible and translating the suffering embedded within modern foodways. These accounts accentuate emotion and the ‘sensing body’ (Longhurst et al., 2008), and build on earlier work that mobilised ethnographic testimony to highlight the central role of place and space in the ordering, obscuring and making kill-able of animals in different food systems (e.g. Vialles, 1994; Taylor and Fraser, 2017).

This work on witnessing-in-place also extends to research on the social injustices and broader ecological harms experienced by certain human bodies within the spaces of animal-free food systems (Harper, 2011; White, 2018). Commentators from beyond vegan studies note how many forms of violence and injustice continue in food supply chains even when animals are removed. Examples include studies of the marginalisation of small-scale avocado farmers in Colombia (Serrano and Brooks, 2019), the ecological and social injustices associated with arable monocultures (Shiva, 1993; Green and Foster, 2005) and the labour and sexual exploitation experienced by migrant workers in horticultural sectors in Europe and the US (Holmes, 2013; Palumbo and Sciurba, 2015). Recognising the spectrum of systemic exploitation within both animal and plant-based foodways has formed a core strand of vegan-anarchist critiques against the ‘vegan-as-consumption’ approach (explored below) to vegan mainstreaming (White, 2018). It also speaks to ongoing efforts to reconcile the focus of animal rights on the welfare of individual animals with environmental ethics which considers the well-being of non-human animals, including livestock, as part of a larger ecosystem of soils, waters and plants. Where some have seen promise in building a more multispecies-focused ethics (Warkentin, 2010), others find their normative positions to be ultimately irreconcilable (Faria and Paez, 2019).

### 3 Corporate veganism

These intersectional and place-based approaches aim to make space for multiple veganisms. But it has been highlighted in discussions on vegetarianism that the inclusivity achieved through an expanded ‘cognitive praxis’ (Morris and Kirwan, 2006) presents a dilemma for those who wish to take a political position on how, and by whom, such movements should be mainstreamed. For example, critics caution against the increasing adoption of health veganism as the commercial pathway through which agri-food corporates market vegan offerings, often as more costly products targeted at wealthier health-conscious consumers. They suggest such trends risk advancing what (White, 2018), 2 terms ‘corporate veganism’ that neutralises the movement’s anti-establishment aim ‘to usher in a more ethical, peaceable, and non-violent world’. These neutralising efforts are apparent in the agri-food industry’s recent uptake of ‘plant-based’ over ‘vegan’ as their favoured product descriptor due to the former being seen as more palatable to a broader consumer base (Sexton, 2018; Clay et al., 2020). Giraud (2021) is similarly critical of this emergent ‘plant-based capitalism’ that has seen individuals and corporate brands exploiting the now fashionable vegan identity without any deeper commitment to systemic ethical living (see also Reed, 2020). These commentators speak for a wider movement in vegan studies calling for urgent critical reflection on this corporatized direction of travel.

These tensions are about who veganism is for, but they also raise questions about who is deemed to be a legitimate agent of change on behalf of the vegan cause, and at what scale. For example, Wrenn champions the power of vegan consumption as a site of individual political resistance, yet cautions against vegan movements operating solely through consumer-based action. She notes the risk of ‘capitalist co-option and moral disillusionment’ (2011, 22) that such approaches can engender, and notes the potential for systemic oppressions to remain unchallenged if consumer action is not complemented by other forms of advocacy, such as policy change and public education. Wrenn also raises a recurring debate about the ‘efficacy and ethical consistency’ (2011, 18) of purchasing vegan products from mainstream brands who derive the majority of their profits from the exploitation of non-human animals, and the potential for veganism to act as a kind of greenwashing that absolves such brands from addressing the intersectional harms that uphold their competitive advantage (Singer, 2017). The work of vegan-anarchist scholars has done much to bring critical focus to the specifically social institutions and political economies that sustain these harms, and to caution against prioritising individual consumptive acts as the only effective means by which to bring about more peaceable living (Dominick, 2015; White, 2015; Milburn, 2016).

Taken together these three strands of vegan studies offer a valuable foundation for critically exploring vegan mainstreaming. However, the critical foci on the spaces of vegan production and veganism as individual practice has tended to cleave to a production-consumption binary. The majority of studies focus primarily on vegan praxis at the individual rather than system level, and of those examining vegan food practices few consider broader agri-food debates on the spatial, political economic and material politics associated with animal-free eating and production networks. A similar production-consumption binary has historically characterised agri-food scholarship, but it has since been challenged by work in food geographies (Lockie and Kitto, 2000; Goodman D., 2004; Kneafsey et al., 2021). This literature helps identify the ‘double-edged sword’ (Smart, 2004) that characterises the ‘vegan-as-consumption’ trend (White, 2018) as one commonly experienced when alternative food networks encounter the market-based, individualistic paradigm of the mainstream (Goodman et al., 2012). Food scholars have begun to highlight the need for greater consideration of these tensions, specifically concerning the potential impacts of transitioning to plant-based operations at the farm and processing levels (Burton, 2019; Lonkila and Kaljonen, 2021; Tziva et al., 2020), and via different production methods (Green and Foster, 2005). We see considerable scope for extending the analytical foundations offered by vegan studies through further engagement with work in food geographies, building on recent interdisciplinary (Morris et al., 2021) and vegan geographies (Hodge et al., forthcoming) research programmes to explore transitions beyond animal-based food systems.

## III Engaging food geographies: Following, alterity and the cultural and material politics of eating

Although veganism remains a marginal concern in food geography, there is copious relevant scholarship within this subfield of geography that explores the potential and pitfalls of mainstreaming what were once alternative ways of farming and eating. Here we identify four key strands from literature in food geographies that extend the foundational interests of vegan studies. These include the conceptual traditions of *following food* through networks and places of its production and consumption, critiquing the *alterity* of alternative food networks, and unpacking the *cultural* and *material politics* of how things become food. We focus primarily on avenues that support further critical work on the specific issue of Big Veganism, but it is hoped the themes we identify can be taken beyond this topic by food geographers to consider contemporary vegan praxis more broadly.

### 1 Following and placing vegan foods

In their manifesto for vegan geographies, Hodge et al. (forthcoming) argue for viewing veganism as an inherently spatial praxis. They make a compelling case for how geographers can extend vegan studies scholarship by attending to the geographies, scales and situatedness of veganism within political, cultural and economic networks, and by tracing unequal spatial patterns in its benefits and impacts. Two tactics stand out for delivering this promise. The first is to engage the conceptual and methodological tradition in geography of ‘following’ food (Cook, 2006) that traces the networks of spaces, people and things connected by production, processing, distribution and consumption. This approach has provided rich accounts of the hidden spaces, practices and political economies that bring foodstuffs from distant geographies to the shelves and menus of mainstream Western retailers. Exemplary work has followed sugar (Mintz, 1986), papayas (Cook, 2004), wine (Kleine, 2008), hot pepper sauce (Cook and Harrison, 2007), coffee (Coles, 2016), wheat (Head and Atchison, 2016) and Fair Trade bananas (Wilson and Jackson, 2016). This approach works in a complementary vein to the autoethnographies of livestock production (Gillespie, 2018; Lockwood, 2018), witnessing the distances travelled by everyday foodstuffs and the lives and experiences of people behind their production and distribution. Following food products works in similarly intimate and political ways to expose the ecological and human exploitation within the commodity chains that underpin modern food systems. Suggested avenues for further geographic work include uncovering the geographies of Big Veganism beyond our focus in this paper on trends in the Global North. For example, by whom and where are supply chains and out-of-sight labour forces being mobilised in order to sustain and grow Big Veganism in the Global North? What impacts are these new and/or existing networks having on local ecologies and communities? To what extent are consumption trends in the Global South pursuing a similar trajectory to those in the North?^2^

A second tactic is to attend to how an *inability *to witness and follow such commodity chains highlights the purposefully hidden injustices that uphold them (Hulme, 2017), for example in work on the heavily guarded interiors of slaughterhouses and factory farms (Taylor and Fraser, 2017). This focus includes the places of food production that have been made invisible due to both their physical location and the political economies of their institutionalised knowledge practices. For example, recent geographic work on soils reveals the knowledge politics that have rendered soil ecosystems as a passive surface to be mapped, owned and worked *upon*, rather than *with* (Krzywoszynska and Marchesi, 2020). Advocating instead for a relational approach to soils, this work offers valuable avenues for vegan-focussed scholarship to literally go deeper into the subterranean places that support Big Veganism. By ‘embedding attentiveness’ (ibid, 199), this conceptual and methodological approach extends critical enquiry into non-human labour and care to the hard-to-access, less sense-able and ‘uncharismatic’ non-human lifeforms within Big Vegan foodways (Puig De la Bellacasa, 2015; Beacham, 2018; Krzywoszynska, 2020).

An attention to place also paradoxically helps to reveal the ‘placelessness’ of some of the world’s most ubiquitous food ingredients (Atchison et al., 2010), including the unspecified starches, gums and preservatives that are now increasingly appearing in mainstream vegan products (Wilson, 2019). This methodological approach brings a critical focus to the broader political economies of vegan mainstreaming that risk locking-in (non)human harms. It also examines the growing ‘displacement’ (Cook and Crang, 1996) of vegan food through its reduction into anonymous industrial ingredients, such as the so-called ‘superfoods’ (Loyer and Knight, 2018) and ‘charismatic nutrients’ (Kimura, 2013) promoted by those making plant-based eating both profitable and palatable.

Following mainstream vegan food also reveals the distinctive split in the recent activities of conventional agribusinesses. While the giants of global food processing and ingredient production (like Tyson Foods and Cargill) have hedged their bets on plant-based trends (Piper, 2019a), resistance has been voiced by both small-scale farmers and the powerful lobby groups that speak for ruminant meat and dairy farming in both the UK and US. The relationships between place and food have become a key battleground within these debates, in which claimed links between livestock, landscapes and producers justify deeply held values about what constitutes ‘real’, ‘simple’ and ‘good’ food (Sexton et al., 2019). Notions of terroir and embeddedness abound in these narratives, reflecting the centrality of place in shaping popular perceptions of the quality and physical makeup of foodstuffs (Mansfield, 2003; Winter, 2003; Morris and Kirwan, 2006; Sexton, 2020). While we note that the care-full embeddedness of conventional livestock production evoked in these narratives does not always reflect realities on the ground, their centrality to a recent backlash against veganism amongst certain farming communities invites further critical exploration.

Some attribute this backlash to a simple resistance to change and an adherence to out-dated values (Hannan, 2020). Without dismissing these factors outright, we suggest a more fruitful avenue is to understand why farmers farm and why some self-identified ‘conscious consumers’ are starting to question the redemptive promises of mainstream vegan products (Piper, 2019b). Work across rural and food geographies has documented the diverse range of personal motivations behind UK farming, especially in the face of the sustained economic losses experienced by many in the sector over recent decades (Food Ethics Council, 2017). For example, Garforth et al. (2006) found that while economic drivers were ranked as important amongst the UK farmers surveyed, a strong sense of stewardship, connection to place and familial heritage were equally, if not more important. There is great scope, we contend, for vegan studies to engage with such work to further understand the place-based hopes, fears and values that are seen to be at stake amongst farming communities with the rise of veganism – both in its ‘Big’ and alternative forms – particularly regarding the physical, economic and cultural shifts in land use and community identity it could catalyse.

Future work following and placing plant-based foods might build on the approaches to witnessing-in-place from vegan studies we reviewed above to explore the experiences of farmers, pickers and other labourers involved in the production networks of Big Veganism. A focus on the grief, fear and anxiety of those working within modern food systems, both animal and ‘post-animal’, would help understand the structural violence that perpetuates modes and scales of farming that are often in conflict with the values of producers. For example, recent reports of the systemic mental health crisis in conventional UK farming (Tasker, 2020), and the emotive stories of livestock-turned-vegan farmers (Sharman, 2019), attest to the uncomfortable and often unwanted lock-ins associated with modern livestock farming (Rebanks, 2020), and necessarily complicate the growing vilification of individual farmers by some within vegan communities for what are system-level failures (Brown, 2019). Moreover, there is little empirical enquiry to date on the experience of those working in the new spaces of post-animal food production, from the supply chains of the latest plant-based analogues to the current laboratories and imagined ‘meat breweries’ of the emerging cellular agriculture sector (Mammoser, 2016; Stephens et al., 2018).

### 2 The alterity of vegan mainstreaming

A defining feature of recent vegan mainstreaming and the high-tech alternative protein products driving these trends has been their framing in media, corporate and advocacy narratives as the ‘better’ alternative for multiple beneficiaries: from animals and ecologies, to individual and societal health (Morris et al., 2018; Sexton et al., 2019). There is a rich literature in food geographies that critically examines comparable claims for the alterity of certain foods, and which can help develop the concerns of intersectional vegan studies with the promises and risks of multiplying veganisms. Work on alternative food networks (AFNs) provides valuable empirical comparisons of the processes inherent to mainstreaming niche food networks and the tensions and contradictions that often arise. Examples of the latter include common tendencies towards ‘unreflexive’ (DuPuis and Goodman, 2005), ‘defensive’ (Winter, 2003) and exclusionary outcomes (Hayes-Conroy and Martin, 2010; Goodman, 2004; Lockie, 2013), and an ultimate failure to meaningfully reimagine, reform or disrupt the models of agri-food capitalism they once stood against (Goodman et al., 2012; Guthman, 2008). This critical work also draws attention to the fascist and elitist heritage of some AFNs, such as the organic movement (Reed, 2001).

For Maye et al. (2007), the label alternative has become empty; a catch-all term for generalised promises of (non)human benefits. In response, attempts have been made to more precisely qualify alterity through discernible metrics and analytical frameworks. For example, Watts et al. (2005) characterise AFNs along a spectrum from ‘weaker’ to ‘stronger’, distinguishing between systems that place emphasis on reimagining either the food product (weaker) or the networks of its production, distribution and broader socioecological services (stronger) (see also Maye et al., 2007; Harris, 2009; Si et al., 2015). Such critiques view AFNs based on individual products as the weaker model for achieving radical change due to their susceptibility to corporate appropriation. According to this analysis, recent trends in vegan mainstreaming have largely followed a weaker model of alterity given their neoliberal, market-based characteristics (White, 2018).

Others have sought to move beyond categorising alterity according to any simplified binary with ‘conventional’ agriculture (Whatmore et al., 2003) and instead aim to acknowledge the heterogeneity, power dynamics and situatedness of different AFNs (Sonnino and Marsden, 2006; Holloway et al., 2007; Wilson, 2013). In different ways this work enables critical yet hopeful appraisals of AFNs based on a ‘reading for difference rather than dominance’ (Gibson-Graham, 2006, xxxi). Such an approach does not seek to dismiss existing criticisms of mainstreamed AFNs and their tendency to reproduce rather than challenge conventional power structures (Guthman, 2008; Lockie, 2013; Slocum and Cadieux, 2015). But neither does it take this failure as the grounds for outright dismissal of the potential for alternative models (like the rise of veganism) to simultaneously enable other political trajectories (Harris, 2009).

Collectively this work on alterity in food geographies offers a reflexive approach for critical research that encourages consideration of what Big Veganism and its associated alternative protein products are claiming to be alternative to. Big Veganism has to date been characterised by explicit alliances with ‘conventional’ agri-food industry, yet it simultaneously claims to be alternative or at least disruptive. The recent acquisitions of vegan start-ups by multinational livestock companies such as Tyson Foods are a case in point. Most of these takeovers have been actively sought by vegan food companies in the name of creating an ‘alternative’ food system that can quickly reach mainstream scales and spaces. Incumbent livestock companies have received widespread praise from many (though not all) vegan scholars and activists who have long opposed conventional animal agri-business (Chiorando, 2020; Friedrich, 2020; Dutkiewicz and Dickstein, 2021). Such evolutions in the way alterity is being operationalised through Big Veganism, and the new ethical, spatial and political economic openings being created offer timely foci for critical food geography (cf. Goodman and Sage, 2014). This research would build on recent geographic work that examines the ontological battlegrounds that have seen the alterity of alternative proteins challenged in legal as well as cultural domains (Jönsson, 2016; Stephens et al., 2019). It also speaks to related studies on the economic geographies of vegan food tech start-ups in Silicon Valley, which are remaking veganism according to the logics and spaces of the high-tech industry (e.g. Mouat et al., 2019; Guthman and Biltekoff, 2020; Jönsson, 2020; Sexton, 2020).

### 3 The cultural politics of vegan eating

The question of how alterity is being mobilised in the mainstreaming of veganism is linked to the question of who bears the responsibility for materialising this transition. White’s (2018) characterisation of veganism-as-consumption identifies how individual consumers and market competition are upheld as the central protagonists in Big Vegan models of food system transition. Food geographers have long questioned this model of political change, in which societal ills are made the responsibility of individuals to solve through their everyday consumption decisions (Guthman, 2003; Goodman, 2004; Johnston, 2008). While not dismissing the collective influence and personal empowerment individual food choice can achieve, many food geographers have highlighted how particularly people of colour, women and lower-income groups disproportionately bear the responsibility and blame for making ‘good’ or ‘bad’ choices (Slocum, 2011; Mansfield, 2012). As such, attention is deflected from system-level issues and the burden of responsibility is shifted away from state and corporate actors (Welch et al., 2018). The governmentalities associated with this model of ‘responsibilising’ the citizen-consumer (Johnston, 2008) have been shown in multiple cases to cultivate problematic and disempowering imaginaries of ‘good’ eaters and ‘good’ eating (Guthman, 2003; Minkoff-Zern, 2014; Gibson and Dempsey, 2013; Sexton, 2018).

In response, Roe (2006a) and others have encouraged relational approaches that challenge the claimed efficacy of individual consumers as agents of systemic change. Undermining the prevalent model of food choice as a set of discrete, free and rational acts, these approaches draw attention to what Goodman (2016) terms the ‘extra-ordinariness’ of food in the repertoire of daily consumptive practices. This work highlights how food is embedded within habituated regimes that shape the pre-cognitive, emotional and multi-sensory dimensions of eating (cf. Hayes-Conroy and Hayes-Conroy, 2008; Jackson et al., 2020). Such work complicates the assertion made by vegan scholars such as Francione (2016, np) that veganism is a choice all persons ‘can make today – right now’. It develops the concerns of intersectional vegan studies scholars to sketch relational ethics that are attuned to the embodied politics of food (Carolan, 2011) and to reveal how networks of spaces, things, visceral experiences and political economic structures shape the capacity for any given individual to enact a care-full ethics in their daily food decisions.

Meanwhile, geographic studies on the cultural politics of food, race, class and gender (Slocum and Saldanha, 2016; Ramírez, 2015; Jones, 2019; Reese, 2019) offer valuable means for deepening the analysis of authenticity and intersectionality in existing vegan studies. For example, reports suggest a notable rise in meat reduction and abstention amongst non-white communities in the US over recent years (McCarthy and Dekoster, 2020). A growing range of non-profit and for-profit ventures, digital movements (#blackvegan) and cultural events (e.g. Black VegFest) have emerged to support and celebrate veganism within black communities in both the UK and US that champions affordable, accessible and nutritionally-rich plant-based foods. Men are also increasingly showing interest in veganism (BUPA, 2018; Henderson, 2018). While research shows many men still feel they need social permission from peers to reduce their meat consumption (Roe, 2018), there has been a discernible shift towards a masculine aesthetic of veganism – achieving its own moniker of ‘heganism’ – that purports to challenge the historic social stigma of plant-based diets as being overtly feminine and nutritionally deficient (cf. Adams, 1990; Asher and Cherry, 2015; Greenebaum and Dexter, 2018). The radical potential of heganism has, however, been criticised for further reinforcing rather than dismantling the problematic identities associated with male veganism (Randall, 2018). The hegan aesthetic has also meant that men increasingly occupy the cultural spaces of mainstream veganism that have predominantly been held by (white) women, and in many of its forms it advances elitist versions of veganism as an often expensive consumptive lifestyle that risks excluding those without the means to buy into costly products and experiences (cf. Giraud, 2021).

Approaches from food geography help critique the political openings and closings enabled by these trends in the cultural politics of food, including those perpetuating problematic racial, class and gendered stereotypes (see Priestley et al., 2016 on the *Thug Kitchen* cookbook). The concurrence of the rise of Big Veganism with the *#MeToo* movement and worldwide Black Lives Matter protests offers a timely moment for exploring the potential shifts in the cultural politics and geographies of vegan representation. Drawing on geographic studies of social movements (e.g. Arenas, 2014; Nicholls, 2007; Pickerill and Chatterton, 2006) and feminist and black food geographies (Garth and Reese, 2020; Hamilton, 2020; Hayes-Conroy and Hayes-Conroy, 2008; Jones, 2019; Reese, 2019), there are also critical questions to ask about how issues of gender, class and racial (in)justice are manifesting within the boardrooms, supply chains and other spaces of Big Veganism.

Finally, food geography is well placed to understand the central place of different media in the cultural politics of vegan mainstreaming. For example, the rise of digital food and lifestyle influencers have enabled the ‘celebritisation’ (Johnston and Goodman, 2015) of vegan activism and promotion, opening up veganism as a new frontier of exposure, controversy and commodification across physical and digital spaces. The proliferation of vegan films and the rise of vegan ‘shock docs’ – such as Cowspiracy, 2014; Forks over knives, 2011; The Game Changers, 2019 – also appears to have played a significant role in recent years in shaping representations of veganism and the planetary issues it aims to solve (Christopher et al., 2018). Further exploration of these vegan media trends could draw on geographic work on the relationship between celebrities, media, food and social activism (Barnes 2017; Phillipov and Goodman, 2017), and the emerging field of digital (food) geographies (Schneider et al., 2017; Ash et al., 2018).

### 4 Materialising veganism: things becoming ‘vegan’ food

The materialist turn (Whatmore, 2006) has led to a renewed focus within food geographies on the physical as well as the cultural and discursive networks that shape everyday food-eater relations. A nascent subfield of this literature has emerged, concerned with ‘the geographies of edibility’ (House, 2018), examining the material processes of how ‘things become food’ (Roe, 2006a); that is, how food is made *as* matter and made *to* matter through claims of betterness and alterity (Evans and Miele, 2012). Many recent studies within this subfield are concerned with foods that promise some form of societal and planetary salvation, such as genetically-modified foods (Roe, 2006b), or the latest generations of alternative proteins including edible insects, cell-cultured meat and plant-based products (House, 2018; Sexton, 2018; Stephens, 2021). This research builds directly on research of historic food trends and the ways animals have been made edible in diverse times and places (Douglas, 1966; Vialles, 1994). It reveals how edibility is contingent, changeable and ‘co-produced by a diverse range of actors’ (House, 2018, 83).

Geographic work on edibility offers valuable conceptual and empirical insights for exploring how an increasingly diverse range of things, from stem cells, to pea protein, to ‘thin air’ (Chowdhury, 2019), are being remade as vegan food. These developments demonstrate the ‘transgressive’ and ‘boundary-crossing’ nature of food (Goodman and Sage, 2014). They invite enquiries into the strategies of edibility formation and how these are reorganising the physical, political economic and ontological dimensions of the food system. This invitation has been taken up by an emerging field of research spanning food geography (Jönsson, 2016; Sexton, 2018; Mouat and Prince, 2018), media studies (Johnson, 2019) and the social studies of science (Stephens, 2013; Jönsson et al., 2019). This work unpacks how alternative proteins transgress the established biological, legal and cultural categories of animal foodstuffs like meat and milk. It reveals how challenging these seemingly stable categories enables broader reimaginings of the spatial and political order of the food system, catalysing collaborations between previously distinct industries (e.g. Big Tech and Big Food) (Guthman and Biltekoff, 2020; Sexton, 2020) and historically opposed ideological positions (e.g. vegan activism and the global livestock industry) (Sexton et al., 2019; Broad, 2019).

Studies of edibility also reveal the distinct visceral politics (Hayes-Conroy and Hayes-Conroy, 2008) that are emerging around these novel foodstuffs. Hedonistic notions of pleasure and good taste are being mobilised through Big Vegan narratives to construct new foodstuffs as the responsible and delicious alternatives to conventional animal-sourced products. Similar trends have been observed in other AFNs like the Slow Food movement (Hayes-Conroy and Martin, 2010), where an appeal to good taste is seen to offer potential for everyday political action against agri-industry. In contrast, Clay et al. (2020) argue that recent efforts to turn plants into milk (or mylk) represent a form of ‘palatable disruption’. Their analysis explores how making vegan food palatable for mainstream tastes involves transforming plants so that they simulate the material properties of meat and dairy: making mylk that pours, creams and froths, and burgers that bleed, sizzle and deliver a chewable ‘mouth feel’. In this way, both the ethical discomfort of desiring animal foods and the sensory discomfort (i.e. texture, bitter tastes) many people associate with plant and fungi-based alternatives are backgrounded. However, the authors caution that consumers are ‘encouraged to care about the environment, health, and animal welfare enough to adopt mylks but to ultimately remain consumers of a commodity food’ (ibid, 2; see also Mylan et al., 2019). Mainstream plant milks as such fit the mould of ‘non-disruptive disruptions’ (Goldstein, 2018) – that is, claiming solutions to systemic problems that merely serve to repair and maintain the capitalist status quo. This analysis raises important questions about who stands to benefit from mass-produced, ersatz commodity vegan foodstuffs.

We see geographic work on visceral politics as a natural complement to the critical feminist turn within vegan studies reviewed above as it draws further attention to how bodies are categorised, represented and made responsible through mainstream vegan discourses. Attention to the visceral politics of Big Veganism invites us to follow these discourses into and through the body, to explore the ‘ways that bodies deal with discourses’ (Hayes-Conroy and Hayes-Conroy 2008, 469) and how sensory experiences are entangled in the corporate and political agendas of Big Veganism (cf. Carolan, 2011). Yet it also offers a conceptual and methodological lens for exploring the hopeful ways in which bodies can resist such co-option. Accounts of dieting and weight loss (Heyes, 2006) and autoethnographic encounters with recent vegan foodstuffs (Sexton, 2016) document the ways in which sensory experiences and expectations can act as a powerful barrier to agri-food industry’s ‘battle for mouths, minds and markets’ (Lang and Heasman, 2015). Mobilising a visceral approach to read for difference over dominance (cf. Gibson-Graham, 2006) helps identify openings to counter some of the problematic material and political economic directions of Big Veganism – for example, using a visceral approach to ask how and by whom the ‘good’ taste of recent vegan foodstuffs is experienced, why some people are resisting them, who is *able* to resist them in favour of ‘real’ animal products or wholefood plant-based ingredients, and who benefits from the particular cultivation of tastes through Big Veganism? More specifically on this latter point, who benefits from mainstream vegan eating remaining largely defined by processed burgers, nuggets and ready meals in place of wholefood plant-based diets?

Understanding edibility and taste as networked beyond the individual also helps extend work in vegan studies on consumer behaviour. For example, Jackson et al.’s (2020) study of taste as something that is publicly shared further highlights the role of supportive environments and peer groups in sustaining commitment to vegan praxis. Moreover, a prominent characteristic of Big Veganism has been the performance of edibility through high-profile and purposefully public tasting events and marketing materials (Stephens and Ruivenkamp, 2016). Collectively these events have worked to position a new generation of vegan foods not only as eatable but also enjoyable (Sexton, 2018), specifically using the public presentation of celebrities, business moguls and food industry figures tasting these novel foods on our behalf (Stephens, 2021). We might think of these highly mediated events as a kind of *visceral witnessing* that works to encourage subjects, through notable conduits, to accept and desire these vegan foodstuffs.

A focus on social context also helps understand the salience of the rise of veganism as a barometer of broader social anxieties. Food geographers have often taken crises as a generative analytic for understanding social norms and practices, in work spanning concerns over global food shortages (Belasco, 2006), contamination and disease (Law and Mol, 2008), to the disconnection of modern food systems (Jackson, 2015). Sexton (2018) argues that the burgeoning demand for alternative proteins represents a materialisation of broader anxieties about the contemporary environmental crisis captured under the zeitgeist label of the Anthropocene. Here vegan foods are normalised as therapeutic edible solutions that ensure planetary salvation, while simultaneously remaining kind to capitalist systems and carnist food cultures.

## IV Vegan food geographies

At the time of writing, the fast-food company Kentucky Fried Chicken (KFC) is in the headlines as the latest global meat behemoth to pin its future to high-tech cell-cultured and plant-based meat alternatives (Young, 2020). The company joins a growing list of livestock, food processing and retail giants who are hedging their bets on animal-free food trends (Piper, 2019a). For some within vegan communities, this latest expression of Big Veganism is to be applauded for offering rapidly scalable transitions away from industrial animal agriculture (Friedrich, 2020). For others, such developments represent uneasy partnerships that neglect the radical ethics and tenets of vegan praxis (White, 2018; Davis, 2019; Giraud, 2021). While these debates continue, what seems increasingly clear is that many agri-food incumbents do not see veganism as a passing consumer fad; as one analyst reported, ‘it’s time has come’ (Hooker, 2018). The cultural, material and political economic effects of its rising popularity are already being felt across the global food system.

Vegan mainstreaming raises a set of timely questions for scholars concerned with the social, animal and environmental consequences of the food system. In this paper we have demonstrated that food geographers are particularly well placed to answer these questions and to interrogate the different vegan food futures that are on the table. We propose the complementary subfield of *vegan food geographies* as a key component of recent agenda-setting research programmes (Morris et al., 2021; Hodge et al., forthcoming) that can critically interrogate contemporary transitions away from animal-based food systems. In this paper we have outlined some preliminary avenues for vegan food geographies research that draw together and develop the common interests and concepts of vegan studies and food geographies. For example, we suggest that conceptual concerns with embeddedness and terroir (Winter 2003) and the methodological tools of following (Cook, 2004) offer potential for further emplacing Big Veganism. These avenues prompt questions of how places feature in the practices and rhetoric of Big Veganism, and where and by whom the promised benefits are being realised. A particular theme we highlight for further critical research is the vision of placelessness in the narratives of some recent products that promise the liberation of animal foodstuffs from the land (and its associated constraints) through the use of stem cell and plant technologies (Sexton et al., 2019; Guthman and Biltekoff, 2020). Geographers need to ask what this imagined liberation means for the future of rural spaces (Burton, 2019) and the people and ecologies they support. Conversely, should we think of Big Veganism as not entirely a vision of placelessness but rather one built around *different* places, such as the synthetic biology lab, the urban farm and the Silicon Valley start-up?

Critical work on race, gender, class and alterity across vegan studies and food geographies (Hayes-Conroy and Hayes-Conroy, 2008; Harper, 2010; Goodman et al., 2012; Reese, 2019) also raises timely questions about the emerging cultural politics of Big Veganism. What has the rise of Big Veganism and its associated ‘alternative’ products done to understandings of alterity and the mainstream? Is it creating new political openings and challenges for doing ‘good’ food (Johnston et al., 2011)? Whose labour and cultural identities support Big Veganism? Who remains visible or hidden in its representations, and who is able or unable to access its benefits and opportunities?

Furthermore, we suggest that new materialist approaches can help develop longstanding concerns with vegan food and eating in cultural studies, while raising a new set of questions. How might taste and edibility be used to understand resistance to veganism by certain producer and consumer groups? What do the processes by which things are being made ‘vegan food’ reveal about the anxieties, cultural politics and political economies of conventional animal foodstuffs? What are the long-term consequences of cultivating vegan tastes through fast-food products and upscaling vegan foodways through the spaces and networks of agri-food incumbents?

We have focussed primarily on themes that are relevant to contemporary trends of vegan mainstreaming, yet there is much scope for vegan food geographies to look beyond these specific developments to other temporalities and contexts of vegan praxis. Likewise, we encourage future work to expand the geographical scope of our analysis beyond Europe and North America. Big Veganism is materialising across global geographies and we are conscious that alternative versions of vegan and vegetarian diets are widespread in other parts of the world, that these diets are changing sometimes as consequences of Northern trends, and that meat and plant consumption is driven by a wider and more diverse set of cultural and political tensions than we can review here. For example, vegetarianism and meat abstinence have been heralded and contested as national virtues in both India and China for very different reasons (Jha, 2004; Staples, 2020). There are diverse vegan food geographies emerging both related to and beyond Big Veganism that are worthy of wider examination.

While by no means exhaustive, our review establishes foundational avenues for a subfield of vegan food geographies that we hope will extend recent critical geographic and social science work on veganism broadly and on the specific issue of its mainstreaming. Zooming out from these suggested research questions, we end the paper with three overarching aims for future vegan food geographies research. First, that future work on Big Veganism attends to the relations between vegan food production and consumption. Our spotlighting of geographic debates on place, moral economies and cultural and material politics offers starting points for considering Big Veganism as more than a product of linear dynamics; it is neither the outcome of a mechanistic chain of production from field to fork, nor the result of consumer-driven trends working backwards down the supply chain. Rather, we encourage work in vegan food geographies to view Big Veganism as embedded within networks of co-constitutive and often contested political, cultural, ethical and material relations. Such a relational approach can, we argue, help to overcome the production-consumption binary that vegan thinking (like agri-food studies before it) has at times perpetuated, and instead encourage a more holistic critique of the implications of veganism’s current mainstreaming. In doing so, vegan food geographies encourages bi-directional lessons between these research fields as a platform to consider how a broader range of bodies, places, privileges and identities are being (re)made through Big Veganism, and to examine the consequences of these dynamics for the power geometries of food and the possibilities for a just and sustainable plant-based food system.

The consequent second aim for vegan food geographies research is to understand Big Veganism as one possible version of mainstreamed veganism. By this we mean to challenge the perceived inevitability of any AFN, including veganism, needing to adopt the scales of operation and political economies of Big Food and Agriculture as the only means of ‘going’ mainstream. Here we look to specific work on alternative food economies and responsible innovation (Rose and Chilvers, 2018; Stilgoe, 2019) that has explored different production systems and scales for maximising accessibility without compromising on original principles and outcomes. For example, the idea of scaling up food production by number (i.e. a large collective of smaller producers) rather than size (a small number of large producers) is one method that has been proposed (Smaje, 2020). Growing movements of intersectional community veganisms and ‘veganic’ (vegan organic) agriculture advance a vision for veganism that is attentive to its spaces and networks of production, land and social reform, and the possibilities of alternative economic models (Harper, 2016; White, 2018; Vegan Organic Network^3^). Broad (2019) and Dutkiewicz (2019) also offer alternative visions for how to support innovation in high-tech vegan food products while embedding principles of food justice and public ownership to mitigate corporate capture and monopolisation. Future work on vegan food geographies is well placed to further explore the potential and limitations of these approaches in the context of veganism and its mainstreaming.

Of course, a core commitment of work in food geography as we have shown has been to make visible the contradictions and harms that can still be perpetuated by even the most care-full ‘alternative’ food networks, many of which become heightened during encounters with the mainstream (Goodman et al., 2012; Reese, 2019). This body of work encourages a critical view of all AFNs as they come to be shaped by the dominant structures and logics of the mainstream. It highlights the distinct ‘politics of the possible’ (Guthman, 2008) that shape the extent to which any AFN, including veganism, can achieve its radical potential as it encounters and becomes shaped by the mainstream – a politics that we highlight here as a necessarily central concern for vegan food geographies research. Yet channelling Gibson-Graham, we should temper a view of the mainstream as homogenous, everywhere and inescapable by ‘reading for difference rather than dominance’ (2006, xxxi). A vegan food geographies approach should remain cautiously open to the political possibilities that Big Veganism is creating. Analysis starts from where we are, treating the existing situation ‘as a (problematic) resource for projects of becoming; a place from which to build something more desirable in the future’ (Gibson-Graham, 2006, xxxii). Taken together, an attention to both the politics of the possible and a reading for difference informs a vegan food geographies approach that considers the surprising and hopeful political openings generated by the trajectories of Big Veganism, while remaining critical of what is overlooked and what might be achieved from other versions of vegan mainstreaming.

Finally, a productive avenue for future work in vegan food geographies would be to further unpack the ‘big’ in Big Veganism. As highlighted in our Introduction, there has been considerable hype in cultural and business commentaries concerning the rise of veganism in mainstream spaces of production and consumption. While the rate of growth in much of these trends has been rapid, the overall picture of veganism in everyday practices remains relatively small in comparison with the continued dominance of global animal food systems. Empirical investigation and interrogation of the ‘big’ in Big Veganism would help to better understand the geographies, temporalities and processes by which recent vegan mainstreaming is occurring, which in turn has important implications for assessing the present and future impacts of transitions away from animal-based food systems.

## Notes

1 For example in 2018, the CEO of global meat company Tyson Foods announced plans to shift the company’s operations from meat production and processing to become a broader ‘producer of proteins’ (Little, 2018). The company has since described itself as ‘The Protein Company^TM^’ in several channels of its corporate branding (cf. White, 2020).

2 Specific thanks to one of our reviewers for helping to develop this set of questions as a critical part of future research on vegan food geographies.

3 See www.veganorganic.net

## References

[bibr1-03091325211051021] Adams CJ (1990) The Sexual Politics of Meat: A Vegetarian Critical Theory. New York, NY: Continuum.

[bibr3-03091325211051021] Arenas I (2014) Assembling the multitude: Material geographies of social movements from Oaxaca to Occupy. Environment and Planning D: Society and Space 32(3): 433–449.

[bibr4-03091325211051021] Ash J Kitchin R Leszczynski A (2018) Digital turn, digital geographies? Progress in Human Geography 42(1): 25–43.

[bibr5-03091325211051021] Asher K Cherry E (2015) Home is where the food is: Barriers to vegetarianism and veganism in the domestic sphere. Journal for Critical Animal Studies 13(1): 66–91.

[bibr6-03091325211051021] Atchison J Head L Gates A (2010) Wheat as food, wheat as industrial substance: Comparative geographies of transformation and mobility. Geoforum 41(2): 236–246.

[bibr7-03091325211051021] Barnes C (2017) Mediating good food and moments of possibility with Jamie Oliver: Problematising celebrity chefs as talking labels. Geoforum 84: 169–178.

[bibr8-03091325211051021] Beacham J (2018) Organising food differently: Towards a more-than-human ethics of care for the Anthropocene. Organization 25(4): 533–549.

[bibr9-03091325211051021] Belasco W (2006) Meals to Come: A History of the Future of Food. Berkeley, CA: University of California Press.

[bibr10-03091325211051021] Broad GM (2019) Plant-based and cell-based animal product alternatives: An assessment and agenda for food tech justice. Geoforum 107: 223–226.

[bibr11-03091325211051021] Brown J (2019) Online abuse and farm protests: the vegans impacting on farmers’ mental health. Sustainable Food Trust. Available at: https://sustainablefoodtrust.org/articles/online-abuse-and-farm-protests-the-vegans-impacting-on-farmers-mental-health/ (accessed 25 January 2021).

[bibr12-03091325211051021] Brueck JF McNeill ZZ (2020) Queer and Trans Voices: Achieving Liberation through Consistent Anti-oppression. London: Sanctuary Publishers.

[bibr13-03091325211051021] BUPA (2018) New research reveals turning 30, 40 or 50 pushes millions of Britons into a lifestyle overhaul. Bupa. Available at: https://www.bupa.com/newsroom/news/zero-effect (accessed 25 January 2021).

[bibr14-03091325211051021] Burton R (2019) The potential impact of synthetic animal protein on livestock production: The new “war against agriculture”. Journal of Rural Studies 68: 33–45.

[bibr15-03091325211051021] Calnan M (2019) Waitrose Launches Vegan Barbeque Lines. The Grocer. Available at: https://www.thegrocer.co.uk/own-label/-waitrose-launches-vegan-barbecue-lines/593029.article (accessed 22 January 2021).

[bibr16-03091325211051021] Carolan MS (2011) Embodied Food Politics. Farnham: Ashgate Publishing Ltd.

[bibr17-03091325211051021] Cherry E (2006) Veganism as a cultural movement: A relational approach. Social Movement Studies 5(2): 155–170.

[bibr18-03091325211051021] Chiorando M (2020) KFC Wins Major Award for its Vegan Burger amid Plant-Based Boom. *Plantbased News*. Available at: https://plantbasednews.org/lifestyle/food/vegan-kfc-wins-award/ (accessed 22 January 2021).

[bibr19-03091325211051021] Chowdury H (2019) Meat' Made from Thin Air? Finland Develops Protein Powder for Human Food Made from Captured CO_2_. The Telegraph. Available at: https://www.telegraph.co.uk/technology/2019/07/08/meat-made-thin-air-finland-develops-protein-powder-human-food/ (accessed 25 January 2021).

[bibr20-03091325211051021] Christopher A Bartkowski JP Haverda T (2018) Portraits of veganism: A comparative discourse analysis of a second-order subculture. Societies 8(3): 55.

[bibr21-03091325211051021] Clay N Sexton AE Garnett T , et al (2020) Palatable disruption: The politics of plant milk. Agriculture and Human Values 37: 1–18. 10.1007/s10460-020-10022-y PMC7644520 33184529

[bibr22-03091325211051021] Cole M Morgan K (2011) Vegaphobia: Discourses of veganism and the reproduction of speciesism in UK national newspapers. The British Journal of Sociology 62(1): 134–153. 21361905 10.1111/j.1468-4446.2010.01348.x

[bibr23-03091325211051021] Coles B (2016). Ingesting places: Embodied geographies of coffee. In: Abbots EJ Lavis A (eds). London: Routledge, pp. 255–271. Why we eat, how we eat: Contemporary encounters between food and bodies

[bibr24-03091325211051021] Cook I (2004) Follow the thing: Papaya. Antipode 36(4): 642–664.

[bibr25-03091325211051021] Cook I Crang P (1996) The world on a plate: Culinary culture, displacement and geographical knowledges. Journal of Material Culture 1(2): 131–153.

[bibr26-03091325211051021] Cook I Harrison M (2007) Follow the Thing: “West Indian Hot Pepper Sauce”. Space and Culture 10(1): 40–63.

[bibr216-03091325211051021] Cook I , et al. (2006) Geographies of food: following. Progress in Human Geography 30(5): 655–666.

[bibr27-03091325211051021] Cook S (2019) Business Ideas for 2019: Plant-Based Foods. *Startups.co.uk*. Available at: https://startups.co.uk/business-ideas/plant-based-foods/ (accessed 22 January 2021).

[bibr28-03091325211051021] Cowspiracy (2014). dir USA: Kip Andersen and Keegan Kuhn, Appian Way Productions. [Film].

[bibr29-03091325211051021] Davis C (2019) Big Meat heats up the plant-based protein market. *Medium*, 29 June 2019. Available at: https://chanapdavis.medium.com/big-meat-heats-up-the-plant-based-protein-market-541553e263c4. (accessed 25 January 2021)

[bibr30-03091325211051021] Dominick B (2015) Anarcho-veganism revisited. In: NocellaII AJ White RJ Cudworth E (eds). Jefferson, NC: McFarland, pp. 23–39. Anarchism and Animal Liberation: Essays on complementary elements of total liberation

[bibr31-03091325211051021] Douglas M (1966) Purity and Danger: An Analysis of Concepts of Pollution and Taboo. London: Routledge & Kegan Paul.

[bibr32-03091325211051021] Doyle J (2016) Celebrity vegans and the lifestyling of ethical consumption. Environmental Communication 10(6): 777–790.

[bibr33-03091325211051021] DuPuis EM Goodman D (2005) Should we go “home” to eat?: Toward a reflexive politics of localism. Journal of Rural Studies 21(3): 359–371.

[bibr34-03091325211051021] Dutkiewicz J (2019). Socialize lab meat. Jacobin. Available at: https://jacobinmag.com/2019/08/lab-meat-socialism-green-new-deal (accessed 8 June 2021).

[bibr35-03091325211051021] Dutkiewicz J Dickstein J (2021) The Ism in Veganism: The Case for a Minimal Practice-based Definition. Food Ethics 6(1): 1–19.

[bibr36-03091325211051021] Evans AB Miele M (2012) Between food and flesh: How animals are made to matter (and not matter) within food consumption practices. Environment and Planning D: Society and Space 30(2): 298–314.

[bibr37-03091325211051021] Faria C Paez E (2019) It’s Splitsville: Why Animal Ethics and Environmental Ethics Are Incompatible. American Behavioral Scientist 63(8): 1047–1060.

[bibr38-03091325211051021] Food Ethics Council (2017) For Whom? Questioning the Food and Farming Research Agenda. London: Food Ethics Council.

[bibr39-03091325211051021] Forchtner B Tominc A (2017) Kalashnikov and cooking-spoon: Neo-Nazism, veganism and a lifestyle cooking show on YouTube. Food, Culture & Society 20(3): 415–441.

[bibr40-03091325211051021] Forks over knives (2011) dir USA: Lee Fulkerson. Film: Monica Beach Media.

[bibr41-03091325211051021] Fox K (2017) Here's Why You Should Turn Your Business Vegan in 2018. Forbes. Available at: https://www.forbes.com/sites/katrinafox/2017/12/27/heres-why-you-should-turn-your-business-vegan-in-2018/ (accessed 22 January 2021).

[bibr42-03091325211051021] Francione GL (2012) Animal welfare, happy meat, and veganism as the moral baseline. In: Kaplan DM (ed). Berkeley: University of California Press, pp. 169–189. The philosophy of food

[bibr43-03091325211051021] Francione GL (2016) The meaning of “The world is VEGAN! If you want it”. The Abolitionist Approach. Available at: https://www.abolitionistapproach.com/14044-2/ (accessed 25 January 2021).

[bibr44-03091325211051021] Friedrich C (2020) I've Not Eaten KFC since the 80s. Its Plant-Based Chicken Nuggets Will Change that. The Guardian. Available at: https://www.theguardian.com/commentisfree/2020/sep/22/ive-not-eaten-kfc-since-the-80s-its-plant-based-chicken-nuggets-will-change-that. (accessed 22 January 2021).

[bibr45-03091325211051021] Fuentes C Fuentes M (2017) Making a market for alternatives: marketing devices and the qualification of a vegan milk substitute. Journal of Marketing Management 33(7–8): 529–555.

[bibr46-03091325211051021] Gaard G (2017) Critical Ecofeminism. Lanham, MD: Lexington Books.

[bibr48-03091325211051021] Gibson-Graham JK (2006) A Postcapitalist Politics. Minneapolis, MN: University of Minnesota Press.

[bibr223-03091325211051021] Garforth C Rehman T McKemey K Yates C Rana RB Green K Wilkinson M Beechener S Hollis K McIntosh L (2006) Research to understand and model the behaviour and motivations of farmers in responding to policy changes (England)). Final report of project EPES0405-17 commissioned by DEFRA. School of Agriculture, Policy and Development, University of Reading, Reading.

[bibr217-03091325211051021] Garth H Reese AM (2020) Black food matters: Racial justice in the wake of food justice. Minneapolis, MN: University of Minnesota Press.

[bibr49-03091325211051021] Gibson KE Dempsey SE (2013) Make good choices, kid: Biopolitics of children’s bodies and school lunch reform in Jamie Oliver’s *Food Revolution*. Children’s Geographies 13(1): 44–58.

[bibr50-03091325211051021] Gillespie K (2018) The Loneliness and Madness of Witnessing: Reflections from a Vegan Feminist Killjoy. In: Probyn-Rapsey F Gruen L (eds). New York, NY: Bloomsbury, pp. 76–85. Animaladies: Gender, Animals, and Madness

[bibr51-03091325211051021] Giraud E (2021) Veganism: Politics, Practice and Theory. London: Bloomsbury.

[bibr52-03091325211051021] Godfray HCJ Aveyard P Garnett T , et al (2018) Meat consumption, health, and the environment. Science 361: eaam5324. 30026199 10.1126/science.aam5324

[bibr53-03091325211051021] Goldstein J (2018) Planetary Improvement: Cleantech Entrepreneurship and the Contradictions of Green Capitalism. Cambridge, MA: MIT Press.

[bibr54-03091325211051021] Good Food Institute (2019) State of the Industry Report: Plant-Based Meat, Eggs, and Dairy. Washington DC: Good Food Institute.

[bibr55-03091325211051021] Goodman D (2004) Rural Europe redux? Reflections on alternative agro‐food networks and paradigm change. Sociologia Ruralis 44(1): 3–16.

[bibr56-03091325211051021] Goodman D DuPuis EM Goodman MK (2012) Alternative Food Networks: Knowledge, Practice and Politics. London: Routledge.

[bibr57-03091325211051021] Goodman MK (2004) Reading fair trade: Political ecological imaginary and the moral economy of fair trade foods. Political Geography 23(7): 891–915.

[bibr58-03091325211051021] Goodman MK (2016) Food geographies I: Relational foodscapes and the busy-ness of being more-than-food. Progress in Human Geography 40(2): 257–266.

[bibr59-03091325211051021] Goodman MK Jaworska S (2020) Mapping digital foodscapes: Digital food influencers and the grammars of good food. Geoforum 117: 183–193.

[bibr60-03091325211051021] Goodman MK Sage C (2014) Food transgressions: Ethics, governance and geographies. In: Goodman MK Sage C (eds). London: Routledge, pp. 1–15. *Food Transgressions: Making Sense of Contemporary Food* Politics

[bibr61-03091325211051021] Green K Foster C (2005) Give peas a chance: Transformations in food consumption and production systems. Technological Forecasting & Social Change 72: 663–679.

[bibr62-03091325211051021] Greenebaum J (2012) Veganism, identity and the quest for authenticity. Food, Culture & Society 15(1): 129–144.

[bibr63-03091325211051021] Greenebaum J Dexter B (2018) Vegan men and hybrid masculinity. Journal of Gender Studies 27(6): 637–648.

[bibr64-03091325211051021] Guthman J (2003) Fast food/organic food: Reflexive tastes and the making of 'yuppie chow. Social & Cultural Geography 4(1): 45–58.

[bibr65-03091325211051021] Guthman J (2008) Neoliberalism and the making of food politics in California. Geoforum 39(3): 1171–1183.

[bibr67-03091325211051021] Guthman J Biltekoff C (2020) Magical Disruption? Alternative Protein and the Promise of De-materialization. *Environment And Planning E: Nature and Space*.

[bibr68-03091325211051021] Hahn LK Bruner MS (2012) Politics on your plate: Building and burning bridges across organic, vegetarian, and vegan discourse. In: Frye J Bruner M (eds). London: Routledge, pp. 55–70. The rhetoric of food: Discourse, materiality and power

[bibr69-03091325211051021] Hamilton AR (2020) The white unseen: On white supremacy and dangerous entanglements in geography. Dialogues in Human Geography 10(3): 299–303.

[bibr70-03091325211051021] Hamilton C (2016) Sex, work, meat: The feminist politics of veganism. Feminist Review 114(1): 112–129.

[bibr71-03091325211051021] Hannan J (2020) Meatsplaining: The Animal Agriculture Industry and the Rhetoric of Denial. Sydney: Sydney University Press.

[bibr72-03091325211051021] Harper AB (2010) Sistah Vegan: Black Female Vegans Speak on Food, Identity, Health, and Society. New York, NY: Lantern Books.

[bibr73-03091325211051021] Harper AB (2011) Vegans of color, racialized embodiment, and problematics of the “exotic”. In: Alkon AH Agyeman J (eds). Cambridge, MA: MIT Press, pp. 221–238. Cultivating food justice: Race, class, and sustainability

[bibr74-03091325211051021] Harper AB (2012) Going beyond the normative white “post-racial” vegan epistemology. In: Forson PW Counihan C (eds). New York, NY: Routledge, pp. 155–174. Taking food public: Redefining foodways in a changing world

[bibr75-03091325211051021] Harper A. B. (2016) Doing veganism differently: Racialized trauma and the personal journey towards vegan healing. In: Hayes-Conroy A Hayes-Conroy J (eds). Abingdon: Routledge, pp. 133–148. Doing Nutrition Differently: Critical Approaches to Diet and Dietary Intervention

[bibr76-03091325211051021] Harris E (2009) Neoliberal subjectivities or a politics of the possible? Reading for difference in alternative food networks. Area 41(1): 55–63.

[bibr77-03091325211051021] Hayes-Conroy A Hayes-Conroy J (2008) Taking back taste: Feminism, food and visceral politics. *Gender, Place and Culture* 15(5Hayes‐Conroy A and Martin DG (2010) Mobilising bodies: visceral identification in the Slow Food movement. Transactions of the Institute of British Geographers 35(2): 461269–473281.

[bibr78-03091325211051021] Head L Atchison J (2016) Ingrained: A Human Bio-Geography of Wheat. London: Routledge.

[bibr79-03091325211051021] Henderson S (2018) Chris Hemsworth's new vegan diet is saving the world. Men’s Health. Available at: https://www.menshealth.com.au/chris-hemsworth-vegan-diet (accessed 25 January 2021).

[bibr80-03091325211051021] Heyes CJ (2006) Foucault goes to weight watchers. Hypatia 21(2): 126–149.

[bibr81-03091325211051021] Hodge P McGregor A Narayan Y , et al (2021) Vegan Geographies: Spaces beyond Violence, Ethics beyond Speciesism.

[bibr82-03091325211051021] Holloway L Kneafsey M Venn L , et al (2007) Possible food economies: a methodological framework for exploring food production–consumption relationships. Sociologia Ruralis 47(1): 1–19.

[bibr83-03091325211051021] Holmes SM (2013) Fresh Fruit, Broken Bodies: Migrant Farmworkers in the United States. Berkeley, CA: University of California Press.

[bibr84-03091325211051021] Hooker L (2018) Tesco Aims to Make Dough Out of Fashion for Veganism, BBC, 10 January 2018. Available at: https://www.bbc.co.uk/news/business-42619887 (accessed 25 January 2021).

[bibr85-03091325211051021] House J (2018) Insects as food in the Netherlands: Production networks and the geographies of edibility. Geoforum 94: 82–93.

[bibr86-03091325211051021] Hulme A (2017) Following the (unfollowable) thing: methodological considerations in the era of high globalisation. cultural Geographies 24(1): 157–160.

[bibr87-03091325211051021] Iacobbo K Iacobbo M (2004) Vegetarian America: A History. Westport, CT: Greenwood Publishing Group.

[bibr88-03091325211051021] Jackson P (2015) Anxious appetites: Food and consumer culture, Vol. 2. London: Bloomsbury Publishing.

[bibr89-03091325211051021] Jackson P Evans D Truninger M , et al (2020) Tasting as a social practice: A methodological experiment in making taste public. Social & Cultural Geography 25: 1–18.

[bibr90-03091325211051021] Jha DN (2004) The Myth of the Holy Cow. New York, NY: Verso Books.

[bibr91-03091325211051021] Johnson H (2019) From 'meat culture' to 'cultured meat': Critically evaluating the contested ontologies and transformative potential of biofabricated animal material on culture and law. M/C Journal 22(2): 1–5.

[bibr92-03091325211051021] Johnston J (2008) The citizen-consumer hybrid: Ideological tensions and the case of Whole Foods Market. Theory and Society 37(3): 229–270.

[bibr93-03091325211051021] Johnston J Goodman MK (2015) Spectacular foodscapes: Food celebrities and the politics of lifestyle mediation in an age of inequality. Food, Culture & Society 18(2): 205–222.

[bibr94-03091325211051021] Johnston J Szabo M Rodney A (2011) Good food, good people: Understanding the cultural repertoire of ethical eating. Journal of Consumer Culture 11(3): 293–318.

[bibr95-03091325211051021] Jones N (2019) Dying to Eat? Black Food Geographies of Slow Violence and Resilience. ACME: An International Journal for Critical Geographies 18(5): 1076–1099.

[bibr96-03091325211051021] Jönsson E (2016) Benevolent technotopias and hitherto unimaginable meats: Tracing the promises of in vitro meat. Social Studies of Science 46(5): 725–748. 28948884 10.1177/0306312716658561

[bibr97-03091325211051021] Jönsson E Linné T McCrow-Young A (2019) Many meats and many milks? The ontological politics of a proposed post-animal revolution. Science As Culture 28(1): 70–97.

[bibr98-03091325211051021] Jönsson E (2020) On breweries and bioreactors: Probing the “present futures” of cellular agriculture. Transactions of the Institute of British Geographers 45(4): 921–936.

[bibr99-03091325211051021] Joy M (2011) Why We Love Dogs, Eat Pigs, and Wear Cows: An Introduction to Carnism. Newburyport, MA: Red Wheel/Weiser.

[bibr100-03091325211051021] Kimura AH (2013) Hidden Hunger: Gender and the Politics of Smarter Foods. Ithaca, NY: Cornell University Press.

[bibr101-03091325211051021] Kleine D (2008) Negotiating partnerships, understanding power: doing action research on Chilean Fairtrade wine value chains. Geographical Journal 174(2): 109–123.

[bibr102-03091325211051021] Kneafsey M Maye D Holloway L , et al (2021) Geographies of Food: An Introduction. London: Bloomsbury.

[bibr103-03091325211051021] Krzywoszynska A (2020) Nonhuman labor and the making of resources: Making soils a resource through microbial labor. Environmental Humanities 12(1): 227–249.

[bibr104-03091325211051021] Krzywoszynska A Marchesi G (2020) Towards a relational materiality of soils. Introduction to the special issue “Conceiving soils and humans in the Anthropocene”. Environmental Humanities 12(1): 190–204.

[bibr105-03091325211051021] Lang T Heasman M (2015) Food Wars: The Global Battle for Mouths, Minds and Markets. London: Routledge.

[bibr106-03091325211051021] Law J Mol A (2008) Globalisation in practice: On the politics of boiling pigswill. Geoforum 39(1): 133–143.

[bibr107-03091325211051021] Leneman L (1999) No animal food: the road to veganism in Britain, 1909-1944. Society & Animals 7(3): 219–228.

[bibr108-03091325211051021] Little A (2018) Tyson Isn’t Chicken. Bloomberg Businessweek. Available at: https://www.bloomberg.com/news/features/2018-08-15/tyson-s-quest-to-be-your-one-stop-protein-shop. (accessed 21 June 2021)

[bibr109-03091325211051021] Lockie S Kitto S (2000) Beyond the farm gate: production‐consumption networks and agri‐food research. Sociologia Ruralis 40(1): 3–19.

[bibr110-03091325211051021] Lockie S (2013) Bastions of white privilege? Reflections on the racialization of alternative food networks. The International Journal of Sociology of Agriculture and Food 20(3): 409–418.

[bibr111-03091325211051021] Lockwood A (2018) Bodily Encounter, Bearing Witness and the Engaged Activism of the Global Save Movement. Animal Studies Journal 7(1): 104–126.

[bibr112-03091325211051021] Longhurst R Ho E Johnston L (2008) Using ‘the body’ as an ‘instrument of research’: Kimch’i and pavlova. Area 40(2): 208–217.

[bibr218-03091325211051021] Lonkila A Kaljonen M (2021) Promises of Meat and Milk Alternatives: An Integrative Literature Review on Emergent Research themes. Agriculture and Human Values 38: 625–639.

[bibr114-03091325211051021] Loyer J Knight C (2018) Selling the “Inca superfood”: Nutritional primitivism in superfoods books and maca marketing. Food, Culture & Society 21(4): 449–467.

[bibr115-03091325211051021] Mammoser G (2016) Why the meat factories of the future will look like breweries. Vice. Available at: https://www.vice.com/en/article/nzk43b/why-the-meat-factories-of-the-future-will-look-like-breweries (accessed 25 January 2021).

[bibr116-03091325211051021] Mansfield B (2003) Spatializing globalization: A “geography of quality” in the seafood industry. Economic Geography 79(1): 1–16.

[bibr117-03091325211051021] Mansfield B (2012) Environmental health as biosecurity: “Seafood choices,” risk, and the pregnant woman as threshold. Annals of the Association of American Geographers 102(5): 969–976.

[bibr118-03091325211051021] Martin E (2019) Veganism, Race, and Soul Food: Evaluating Reproductions of Race in Vegan Spaces. Wellesley College, US: Thesis.

[bibr120-03091325211051021] Maurer D (2002) Vegetarianism: Movement or Moment? Philadelphia, PA: Temple University Press.

[bibr121-03091325211051021] Maye D Kneafsey M Holloway L (2007) Introducing alternative food geographies. In: Maye D Holloway L Kneafsey M (eds). Oxford: Elsevier, pp. 1–20.Alternative food geographies: representation and practice

[bibr122-03091325211051021] McCarthy J Dekoster S (2020) Nearly one in four in U.S. have cut back on eating meat. Gallup. Available at: https://news.gallup.com/poll/282779/nearly-one-four-cut-back-eating-meat.aspx (accessed 25 January 2021).

[bibr123-03091325211051021] Michail N (2018) Unilever Accelerates its ‘plant-Based Journey’ with Vegetarian Butcher Buyout. *FoodNavigator.Com*. Available at: https://www.foodnavigator.com/Article/2018/12/20/Unilever-accelerates-its-plant-based-journey-with-Vegetarian-Butcher-buyout (accessed 22 January 2021).

[bibr124-03091325211051021] Milburn J (2016) Animal rights and food: Beyond Regan, beyond vegan. In: Rawlinson M Ward C (eds). London: Routledge, pp. 284–294. The Routledge Handbook of Food Ethics

[bibr127-03091325211051021] Minkoff‐Zern LA (2014) Knowing “good food”: Immigrant knowledge and the racial politics of farmworker food insecurity. Antipode 46(5): 1190–1204.

[bibr128-03091325211051021] Mintz SW (1986) Sweetness and Power: The Place of Sugar in Modern History. New York, NY: Penguin Books.

[bibr129-03091325211051021] Monaghan A (2019) Greggs’ Vegan Sausage Rolls Fuel Profit Boom. The Guardian. Available at: https://www.theguardian.com/business/2019/may/14/greggs-vegan-sausage-rolls-fuel-profit-boom (accessed 22 January 2021).

[bibr130-03091325211051021] Morris C Kirwan J (2006) Vegetarians: uninvited, uncomfortable or special guests at the table of the alternative food economy? Sociologia Ruralis 46(3): 192–213.

[bibr131-03091325211051021] Morris C Mylan J Beech E (2018) Substitution and food system de-animalisation: the case of non-dairy milk. International Journal of Sociology of Agriculture and Food 25(1): 490–506.

[bibr132-03091325211051021] Morris C Kaljonen M Aavik K , et al (2021) Priorities for social science and humanities research on the challenges of moving beyond animal-based food systems. Humanities and Social Sciences Communications 8(1): 1–12.

[bibr133-03091325211051021] Mouat MJ Prince R (2018) Cultured meat and cowless milk: On making markets for animal-free food. Journal of Cultural Economy 11(4): 315–329.

[bibr134-03091325211051021] Mouat MJ Prince R Roche MM (2019) Making value out of ethics: the emerging economic geography of lab-grown meat and other animal-free food products. Economic Geography 95(2): 136–158.

[bibr135-03091325211051021] Mylan J Morris C Beech E , et al (2019) Rage against the regime: niche-regime interactions in the societal embedding of plant-based milk. Environmental Innovation and Societal Transitions 31: 233–247.

[bibr136-03091325211051021] Nicholls WJ (2007) The geographies of social movements. Geography Compass 1(3): 607–622.

[bibr137-03091325211051021] Nguyen S (2017) The Rise of Vegan Culture. Harvard Magazine. Available at: https://harvardmagazine.com/2017/07/the-rise-of-vegan-culture (accessed 22 January 2021).

[bibr138-03091325211051021] Oliver C (2020) Beyond-human research: Negotiating silence, anger & failure in multispecies worlds. Emotion, Space and Society 35: 100686.

[bibr139-03091325211051021] Palumbo L Sciurba A (2015) Vulnerability to forced labour and trafficking: The case of Romanian women in the agricultural sector in Sicily. Anti-Trafficking Review 5: 89–108.

[bibr140-03091325211051021] Parker J (2018) The Year of the Vegan. The World in 2019. The Economist.

[bibr141-03091325211051021] PBFA (2018) Retail Sales Data 2018. Plant Based Foods Association. Available at: https://www.plantbasedfoods.org/marketplace/retail-sales-data-2018/ (accessed 17 August 2021).

[bibr142-03091325211051021] Pendergrast N (2016) Environmental concerns and the mainstreaming of veganism. In: Raphaely T Marinova D (eds). Hershey, PA: IGI Global, pp. 106–122. Impact of Meat Consumption on Health and Environmental Sustainability

[bibr143-03091325211051021] Phillipov M Goodman MK (2017) The celebrification of farmers: celebrity and the new politics of farming. Celebrity Studies 8(2): 346–350.

[bibr219-03091325211051021] Pickerill J Chatterton P (2006) Notes towards autonomous geographies: creation, resistance and self-management as survival tactics. Progress in human geography 30(6): 730–746.

[bibr144-03091325211051021] Piper K (2019a) The unlikely partnership that might decide the future of meat. Vox. Available at: https://www.vox.com/future-perfect/2019/3/22/18273892/tyson-vegan-vegetarian-lab-meat-climate-change-animals (accessed 25 January 2021).

[bibr145-03091325211051021] Piper K (2019b) Meatless meat is becoming mainstream—and it’s sparking backlash. Vox. Available at: https://www.vox.com/future-perfect/2019/10/7/20880318/meatless-meat-mainstream-backlash-impossible-burger (accessed 25 January 2021).

[bibr146-03091325211051021] Polish J (2016) Decolonizing veganism: On resisting vegan whiteness and racism. In: Castricano J Simonsen R (eds). Cham: Palgrave Macmillan, pp. 373–391. Critical Perspectives on Veganism. The Palgrave Macmillan Animal Ethics Series

[bibr147-03091325211051021] Poore J Nemecek T (2018) Reducing food’s environmental impacts through producers and consumers. Science 360(6392): 987–992. 29853680 10.1126/science.aaq0216

[bibr148-03091325211051021] Priestley A Lingo SK Royal P (2016) “The worst offense here is the misrepresentation”: *Thug Kitchen* and contemporary vegan discourse. In: Castricano J Simonsen R (eds). Cham: Palgrave Macmillan, pp. 349–371. Critical Perspectives on Veganism. The Palgrave Macmillan Animal Ethics Series

[bibr149-03091325211051021] Puig de la Bellacasa M (2015) Making time for soil: Technoscientific futurity and the pace of care. Social Studies of Science 45(5): 691–716. 26630817 10.1177/0306312715599851

[bibr150-03091325211051021] Ramírez MM (2015) The elusive inclusive: Black food geographies and racialized food spaces. Antipode 47(3): 748–769.

[bibr151-03091325211051021] Randall TE (2018) Heganism. Between the Species 22(1): 214–235.

[bibr152-03091325211051021] Rebanks J (2020) English Pastoral: An Inheritance. London: Penguin Books.

[bibr153-03091325211051021] Reed M (2001) Fight the future! How the contemporary campaigns of the UK organic movement have arisen from their composting of the past. Sociologia Ruralis 41(1): 131–145.

[bibr154-03091325211051021] Reed S (2020) “Part-time” Vegans Are on the Rise–And “Real” Vegans Are Mad about it. InStyle. 3 March 2020. Available at: https://www.instyle.com/beauty/health-fitness/vegans-mad-part-time-vegans (accessed 22 January 2021).

[bibr155-03091325211051021] Reese AM (2019) Black Food Geographies: Race, Self-Reliance, and Food Access. Washington, DC: Chapel HillUNC Press Books.

[bibr156-03091325211051021] Regan T (1983) The Case for Animal Rights. Berkeley, CA: The University of California Press.

[bibr157-03091325211051021] Roe EJ (2006a) Things becoming food and the embodied, material practices of an organic food consumer. Sociologia Ruralis 46(2): 104–121.

[bibr158-03091325211051021] Roe EJ (2006b) Material connectivity, the immaterial and the aesthetic of eating practices: an argument for how genetically modified foodstuff becomes inedible. Environment and Planning A 38(3): 465–481.

[bibr159-03091325211051021] Roe EJ (2018) Sun’s Out, Buns Out: Exploring the alfresco ritual of meat, fire, man’s work, and sustainability. Global Food Security. Available at: https://www.foodsecurity.ac.uk/blog/suns-out-buns-out-exploring-the-alfresco-ritual-of-meat-fire-mans-work-and-sustainability/ (accessed 25 January 2021).

[bibr160-03091325211051021] Rose DC Chilvers J (2018) Agriculture 4.0: Broadening responsible innovation in an era of smart farming. Frontiers in Sustainable Food Systems 2(87): 1–7.

[bibr161-03091325211051021] Schneider T Eli K Dolan C , et al (eds) (2017). London: Routledge. Digital food activism

[bibr162-03091325211051021] Serrano A Brooks A (2019) Who is left behind in global food systems? Local farmers failed by Colombia’s avocado boom. Environment and Planning E: Nature and Space 2(2): 348–367.

[bibr220-03091325211051021] Sexton A (2016) Alternative proteins and the (non) stuff of “meat”. Gastronomica: The Journal of Critical Food Studies 16(3): 66–78.

[bibr163-03091325211051021] Sexton AE (2018) Eating for the post‐Anthropocene: Alternative proteins and the biopolitics of edibility. Transactions of the Institute of British Geographers 43(4): 586–600.

[bibr164-03091325211051021] Sexton AE (2020) Food as Software: Place, Protein, and Feeding the World Silicon Valley-style. Economic Geography. 10.1080/00130095.2020.1834382 PMC7617018 39650754

[bibr165-03091325211051021] Sexton AE Garnett T Lorimer J (2019) Framing the future of food: The contested promises of alternative proteins. Environment and Planning E: Nature and Space 2(1): 47–72. 10.1177/2514848619827009 PMC6989034 32039343

[bibr166-03091325211051021] Sharman J (2019) Sheep Farmer Felt So Guilty on Way to Slaughterhouse He Drove Lambs to Sanctuary and Became Vegetarian. Independent. Available at: https://www.independent.co.uk/news/uk/home-news/sheep-farmer-vegetarian-lambs-sanctuary-slaughter-meat-industry-dairy-devon-a8754056.html (accessed 25 January 2021).

[bibr167-03091325211051021] Shiva V (1993) Monocultures of the Mind: Perspectives on Biodiversity and Biotechnology. London: Zed Books.

[bibr168-03091325211051021] Shoup ME (2019) Euromonitor: What’s the driving force behind plant-based eating? Foodnavigator.com. Available at: https://www.foodnavigator-usa.com/Article/2019/05/17/Euromonitor-What-s-the-driving-force-behind-plant-based-eating (accessed 22 January 2021).

[bibr169-03091325211051021] Si Z Schumilas T Scott S (2015) Characterizing alternative food networks in China. Agriculture and Human Values 32(2): 299–313.

[bibr170-03091325211051021] Singer R (2017) Neoliberal backgrounding, the Meatless Monday Campaign, and the rhetorical intersections of food, nature, and cultural identity. Communication, Culture and Critique 10(2): 344–364.

[bibr171-03091325211051021] Slocum R (2011) Race in the study of food. Progress in Human Geography 35(3): 303–327.

[bibr172-03091325211051021] Slocum R Cadieux KV (2015) Notes on the practice of food justice in the US: understanding and confronting trauma and inequity. Journal of Political Ecology 22(27): 27–52.

[bibr173-03091325211051021] Slocum R Saldanha A (eds) (2016). London: Routledge.Geographies of race and food: Fields, bodies, markets

[bibr221-03091325211051021] Smaje C (2020) A small farm future: Making the case for a society built around local economies, self-provisioning, agricultural diversity and a shared earth. London: Chelsea Green Publishing.

[bibr174-03091325211051021] Smart A (2004) Adrift in the mainstream: Challenges facing the UK vegetarian movement. British Food Journal 106(2): 79–92.

[bibr175-03091325211051021] Smithers R (2019) Veganuary Ends on Record High with 250,000 Participants. The Guardian. Available at: https://www.theguardian.com/lifeandstyle/2019/jan/31/veganuary-record-high-participants-plant-based (accessed 22 January 2021).

[bibr176-03091325211051021] Sneijder P Te Molder HF (2004) ‘Health should not have to be a problem’: Talking health and accountability in an internet forum on veganism. Journal of Health Psychology 9(4): 599–616. 15231059 10.1177/1359105304044046

[bibr177-03091325211051021] Sonnino R Marsden T (2006) Beyond the divide: rethinking relationships between alternative and conventional food networks in Europe. Journal of Economic Geography 6(2): 181–199.

[bibr178-03091325211051021] Spencer C (1996) The Heretic's Feast: A History of Vegetarianism. Lebanon, NH: UPNE.

[bibr179-03091325211051021] Staples J (2020) Sacred Cows and Chicken Manchurian: The Everyday Politics of Eating Meat in India. Seattle, WA: University of Washington Press.

[bibr180-03091325211051021] Stephens N (2013) Growing meat in laboratories: The promise, ontology, and ethical boundary-work of using muscle cells to make food. Configurations 21(2): 159–181.

[bibr181-03091325211051021] Stephens N Ruivenkamp M (2016) Promise and ontological ambiguity in the in vitro meat imagescape: From laboratory myotubes to the cultured burger. Science As Culture 25(3): 327–355. 27695202 10.1080/09505431.2016.1171836 PMC5022697

[bibr182-03091325211051021] Stephens N Di Silvio L Dunsford I , et al (2018) Bringing cultured meat to market: Technical, socio-political, and regulatory challenges in cellular agriculture. Trends in Food Science and Technology 78: 155–166. 30100674 10.1016/j.tifs.2018.04.010 PMC6078906

[bibr183-03091325211051021] Stephens N Sexton AE Driessen C (2019) Making sense of making meat: Key moments in the first 20 years of tissue engineering muscle to make food. Frontiers in Sustainable Food Systems 3(45): 1–16. 10.3389/fsufs.2019.00045 PMC7611147 34250447

[bibr184-03091325211051021] Stephens N (2021) Join our team, change the world: Edibility, producibility and food futures in cultured meat company recruitment videos. Food, Culture & Society 11: 1–17. 10.1080/15528014.2021.1884787 PMC8842710 35177960

[bibr222-03091325211051021] Stilgoe J (2019) Who’s driving innovation? New technologies and the collaborative state. Cham, Switzerland: Springer Nature Switzerland.

[bibr185-03091325211051021] Stuckler D Nestle M (2012) Big Food, Food Systems, and Global Health. PLoS Med 9(6): e1001242. 22723746 10.1371/journal.pmed.1001242 PMC3378592

[bibr186-03091325211051021] Tasker J (2020) Farming faces mental health crisis, warns charity. Farmers Weekly. Available at: https://www.fwi.co.uk/business/business-management/health-and-safety/farming-faces-mental-health-crisis-warns-charity (accessed 25 January 2021).

[bibr187-03091325211051021] Taylor N Fraser H (2017) *Slaughterhouses:* The Language of Life, the Discourse of Death. In: Maher J Pierpoint H Beirne P (eds). London: Palgrave Macmillan. The Palgrave International Handbook of Animal Abuse Studies

[bibr188-03091325211051021] The Game Changers (2019) Louie Psihoyos. Dir. USA: OPS Productions and ReFuel Productions. [Film].

[bibr189-03091325211051021] Tulloch LK (2018) An Auto-ethnography of Anti-dairy Vegan Activism in New Zealand. Animal Studies Journal 7(2): 180–214.

[bibr190-03091325211051021] Twine R (2014a) Vegan killjoys at the table: Contesting happiness and negotiating relationships with food practices. Societies 4(4): 623–639.

[bibr191-03091325211051021] Twine R (2014b) Ecofeminism and veganism: Revisiting the question of universalism. In: Adams CJ Gruen L (eds). Ecofeminism: Feminist intersections with other animals and the earth. New York: Bloomsbury, 191–209.

[bibr192-03091325211051021] Tziva M Negro SO Kalfagianni A , et al (2020) Understanding the protein transition: The rise of plant-based meat substitutes. Environmental Innovation and Societal Transitions 35: 217–231.

[bibr193-03091325211051021] Véron O (2016) From Seitan Bourguignon to Tofu Blanquette: Popularizing veganism in France with food blogs. In: Castricano J Simonsen R (eds). Cham: Palgrave Macmillan, pp. 287–305. Critical Perspectives on Veganism. The Palgrave Macmillan Animal Ethics Series

[bibr194-03091325211051021] Vialles N (1994) Animal to Edible. Cambridge: Cambridge University Press.

[bibr195-03091325211051021] Waldstein A (2016) Studying the body in Rastafari rituals: Spirituality, embodiment and ethnographic knowledge. Journal for the Study of Religious Experience 2(1): 71–86.

[bibr196-03091325211051021] Warkentin T (2010) Interspecies etiquette: An ethics of paying attention to animals. Ethics & the Environment 15(1): 101–121.

[bibr197-03091325211051021] Warkentin T (2012) Must every animal studies scholar be vegan? Special Issue: Animal Others, Invited Symposium: Feminists Encountering Animals. Hypatia: Journal of Feminist Philosophy 27(3): 499–504.

[bibr198-03091325211051021] Watts DCH Ilbery B Maye D (2005) Making reconnections in agro-food geography: Alternative systems of food provision. Progress in Human Geography 29(1): 22–40.

[bibr199-03091325211051021] Welch D Swaffield J Evans D (2018) Who’s responsible for food waste? Consumers, retailers and the food waste discourse coalition in the United Kingdom. Journal of Consumer Culture: 13: 1–21.

[bibr200-03091325211051021] Werkheiser I (2013) Domination and consumption: An examination of veganism, anarchism, and ecofeminism. PhaenEx 8(2): 161–184.

[bibr201-03091325211051021] Whatmore S (2006) Materialist returns: practising cultural geography in and for a more-than-human world. Cultural Geographies 13(4): 600–609.

[bibr202-03091325211051021] Whatmore S Stassart P Renting H (2003) What's alternative about alternative food networks? Environment and Planning A 35: 389–391.

[bibr203-03091325211051021] White N (2020) The Protein Company^TM^: Meeting the world’s appetite. Tyson Foods: The Feed Blog. Available at: https://thefeed.blog/2020/02/26/tyson-foods-protein-company-feeding-the-world/ (accessed 21 June 2021).

[bibr204-03091325211051021] White R (2015) Animal geographies, anarchist praxis, and critical animal studies. In: Gillespie K Collard R-C (eds). London: Routledge, pp. 31–47. Critical animal geographies: Politics, intersections, and hierarchies in a multispecies world

[bibr205-03091325211051021] White R Cudworth E (2014) Taking It to the Streets: Challenging Systems of Domination from Below. Counterpoints 448: 202–219.

[bibr207-03091325211051021] White RJ (2017) Rising to the challenge of capitalism and the commodification of animals: Post-capitalism, anarchist economies and vegan praxis. In: Nibert D (ed), Westport, CT: Praeger.Animal Oppression and Capitalism

[bibr206-03091325211051021] White RJ (2018) Looking backward/moving forward. Articulating a “Yes, BUT!” response to lifestyle veganism, and outlining post-capitalist futures in critical veganic agriculture. EuropeNow 20: 1–13.

[bibr208-03091325211051021] Wilson AD (2013) Beyond alternative: Exploring the potential for autonomous food spaces. Antipode 45(3): 719–737.

[bibr209-03091325211051021] Wilson B (2019) The trouble with fake meat. The Guardian. Available at: https://www.theguardian.com/food/2019/jan/27/the-trouble-with-fake-meat-beetroot-burgers-food-substitutes (accessed 25 January 2021).

[bibr210-03091325211051021] Wilson M Jackson P (2016) Fairtrade bananas in the Caribbean: Towards a moral economy of recognition. Geoforum 70: 11–21.

[bibr211-03091325211051021] Winter M (2003) Embeddedness, the new food economy and defensive localism. Journal of Rural Studies 19(1): 23–32.

[bibr212-03091325211051021] Wrenn CL (2011) Resisting the globalization of speciesism: Vegan abolitionism as a site for consumer-based social change. Journal for Critical Animal Studies 9(3): 9–27.

[bibr213-03091325211051021] Wrenn CL (2017) Trump veganism: A political survey of American vegans in the era of identity politics. Societies 7(4): 32–45.

[bibr214-03091325211051021] Wright L (2015) The Vegan Studies Project: Food, Animals, and Gender in the Age of Terror. Athens, GA: University of Georgia Press.

[bibr215-03091325211051021] Young S (2020) ‘Meat of the Future’: KFC Is Developing the World’s First Lab-Grown Chicken Nuggets. Independent. Available at: https://www.independent.co.uk/life-style/food-and-drink/kfc-lab-grown-chicken-nuggets-biomeat-3d-bioprinting-russia-a9629671.html (accessed 25 January 2021).

